# Protrusion of a rod into the spinal canal 10 years after segmental lumbar spine surgery

**DOI:** 10.1097/MD.0000000000006425

**Published:** 2017-03-24

**Authors:** Siyi Cai, Xiangyi Kong, Chengrui Yan, Yipeng Wang, Xueshuai Wan, Jialu Zhang, Guixing Qiu, Keyi Yu

**Affiliations:** a Department of Orthopaedic Surgery; b Department of Neurosurgery, Peking Union Medical College Hospital, Peking Union Medical College and Chinese Academy of Medical Sciences, Beijing, China; c Department of Anesthesia, Critical Care and Pain Medicine, Massachusetts General Hospital, Harvard Medical School, Harvard University, MA.

**Keywords:** rod protruding, segmental lumbar spine surgery, spinal canal

## Abstract

The objective of this article is to report an unusual case of a spinal rod that protruded into the spinal canal after lumbar spine surgery.

Only 4 cases of spinal rod migration with protrusion into the spinal canal have been reported. This is the first report of a case involving the use of posterior low lumbar segmental instrumentation with a screw–rod system. The left side of the rod gradually migrated and finally protruded into the canal and compressed the cord.

A 60-year-old woman presented with pain and numbness of the posterior aspect of the left leg after a long-distance walk. Intermittent claudication became worse, and she developed pain and numbness in the perineal region. An x-ray showed that the left side of a spinal rod among the segmental spinal instruments that had been placed 10 years previously had protruded into the spinal canal.

We removed the rod and decompressed the canal at the level of L5-S1. The patient became totally asymptomatic.

Rods used as spinal instrumentation have the possibility of protruding into the spinal canal and endangering the nervous system. Long-term follow-up with radiological examinations should be conducted upon completion of spinal operations conducting using instrumentation.

## 1 Introduction

Implant failure after spinal surgery sometimes occurs; however, protrusion of rods into the spinal canal is rare. We herein report an unusual case involving a rod from a segmental lumbar spinal surgery that gradually migrated and finally protruded into the lumbar spinal canal and compressed the cauda equina. Written informed consent was obtained from the patient for publication of this article. A copy of the written consent is available for review by the editors of MEDICINE. Because this article does not involve any human or animal trials, there is no need to conduct special ethic review and the ethical approval is not necessary.

## 2 Case presentation and analysis

**Figure 1:**
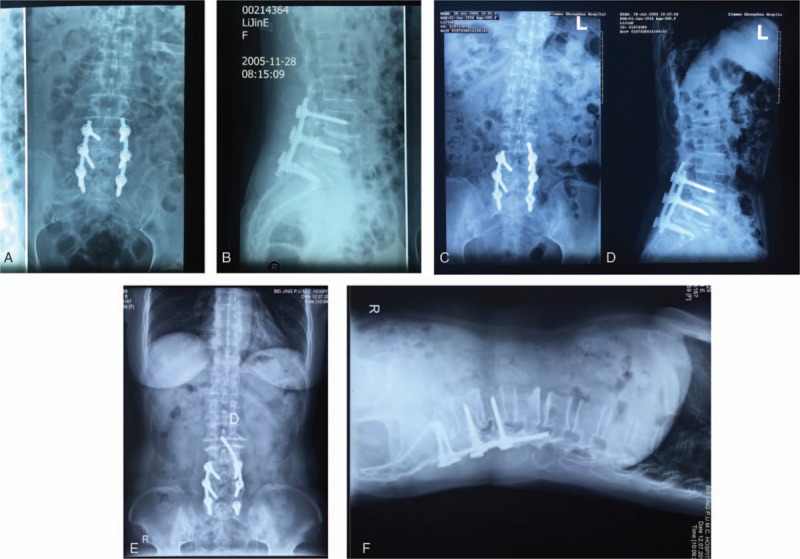
(A, B) Anterior and lateral views of the lumbar spine with lumbar instrumentation 1 day postoperatively. (C, D) Anterior and lateral views of the lumbar spine 1 year postoperatively. The left rod had migrated. (E, F) Anterior and lateral views of the lumbar spine. Eleven years later, the migrated rod protruded into the spinal canal.

A 60-year-old woman underwent lumbar spinal surgery with instrumentation twice at almost the same site nearly 10 years before presentation to our hospital. These 2 operations were performed at another institution. The patient was diagnosed with isthmic spondylolisthesis (L4 grade II, L5 grade II) and spinal stenosis (L4-S1 and L4-L5; worse at L4-L5) with lumbar disc herniation (L4/5, L5/S1) on the left side. Using the posterior approach, the L4-L5 spinal segments were decompressed and the herniated disc was removed from the left side of the lamina window between L4 and L5. The L4 and L5 lumbar vertebrae were reduced and fixed using 2 rods and 6 monoaxial pedicle screws (Moss Miami SI System; DePuy), which were placed in L4-L5 and S1 bilaterally. Interbody fusion of L4-L5 and S1 was performed with allograft bone, and laminal fusion was performed from the right side of L4-L5 and extended to the S1 lamina. The posterolateral left leg claudication and low back pain disappeared. One month later, however, the patient developed posteriorly radiating pain in the left leg because of S1 nerve compression caused by a piece of the interbody bone graft located at L5-S1. A second operation was performed laterally. The left sides of the L5 and S1 laminae were partially resected, and the small piece of herniated bone was successfully removed. The patient's leg pain then disappeared (Fig. 1A and B). One year later, the left rod had migrated slightly caudally. The patient chose not to undergo another operation to remove the implant because she was asymptomatic (Fig. 1C and D).

**Figure 2:**
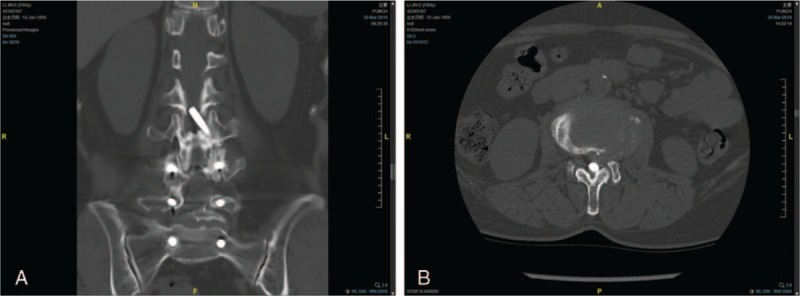
Computed tomography shows (A) coronal reconstruction and (B) an axial section shows intraspinal migration of the left rod.

**Figure 3:**
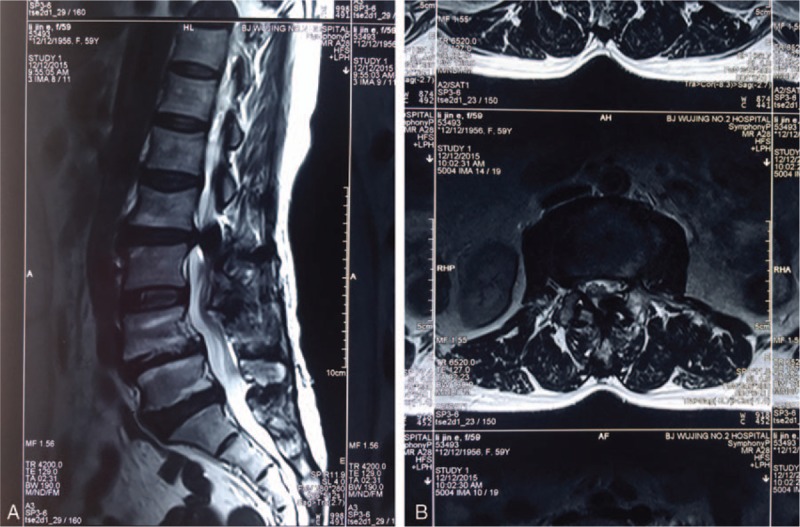
Magnetic resonance imaging shows (A) sagittal reconstruction and (B) an axial section shows intraspinal migration of the left rod.

One year before presentation to our hospital, the patient developed pain and numbness of the posterior aspect of the left leg after a long-distance walk. The intermittent claudication became worse, and the distance that she was able to walk gradually shortened. One month before presentation, she developed pain and numbness in the perineal region when sitting for a long period of time. Plain films showed that the rod had shifted a long distance since last examined 10 years previously (Fig. 1E and F). Computed tomography (CT; Fig. 2) and magnetic resonance imaging (MRI; Fig. 3) showed that the left rod had migrated and protruded into the spinal canal. This caused compression of the dural sac at L2-L3.

**Figure 4:**
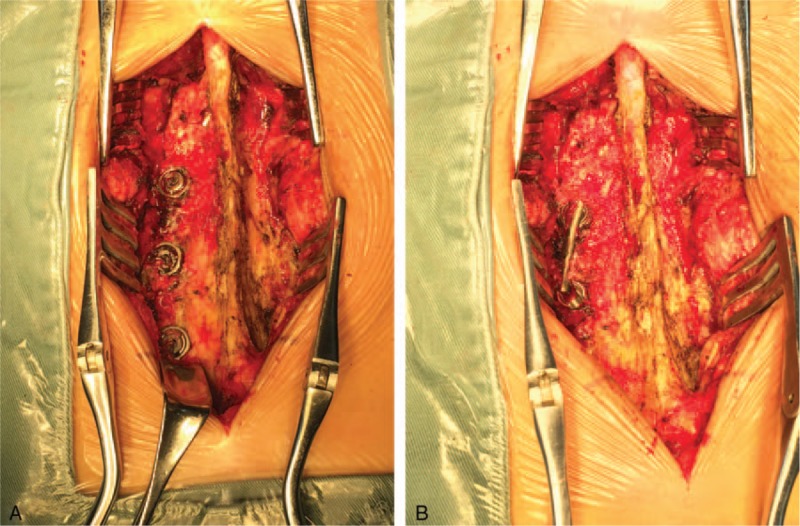
Intraoperative view showing (A) the rod protruding into the canal and a fusion mass covering the rod; (B) the rod has almost slid out of the left L5 screw.

We removed and replace the rod with 3 polyaxial screws on the left side. We performed transforaminal lumbar interbody fusion and decompression of the L5-S1 recess area from the posterior approach on the left side (Fig. 4). Specimens were obtained for bacteriological culture from the 3 screw holes and tissue near the rod on the left side. We found no evidence of infection.

## 3 Discussion

Surgical treatment for lumbar spondylolisthesis and secondary stenosis is performed by the posterior approach. When an isthmic defect is present, interbody fusion is more reliable. Posterior laminal fusion may be dangerous, and implant failure may be caused by a lack of fusion. However, migration of a rod that subsequently penetrates the lumbar canal and causes nerve deficits is rare.

Only 4 cases of intracanal rod migration have been reported. In 1993, Quint and Salton^[1]^ described a young man who developed an L1 vertebral fracture dislocation and was treated with anterior and posterior fusion surgery. The Luque rods that had been used during the fusion surgery migrated into the spinal canal from the T10 laminectomy defect 10 years after the index operation. In 2003, Tribus and Garvey^[2]^ presented a case involving a Cotrel–Dubousset rod that migrated through a thoracic laminar defect because of progressive bone erosion. An etiological examination showed active *Propionibacterium acnes* and *Staphylococcus epidermidis* infection. In this case, the authors attributed the erosion to a mechanical etiology. Although possible infectious pathogenic bacteria were found on both sides of the implants, erosion was only found on the convex side. In 2016, another report described a 16-year-old boy with adolescent idiopathic scoliosis who underwent posterior correction. Instrumented fusion was performed 8 years previously. The authors concluded that the cause of the laminar erosion was a low-grade chronic infection^[3]^. The most recent case was reported in 2017 and involved a 13-year-old boy with spastic cerebral palsy. Multilevel Smith–Petersen osteotomies were performed, and severe kyphoscoliosis was corrected. Instrumented fixed fusion was performed from T1-L5, and 2 intrasacral rods were applied. Five years later, the patient complained of progressive paresthesia and loss of lower limb motor function. Radiological examinations showed that both rods had migrated into the spinal canal. The authors considered that the migration was due to the patient's muscular disorder^[4]^.

In these previous 4 cases, metallosis and late infection were attributed to rod migration. In the present case, we assumed that improper manipulation and the design of the pedicle screw structure were the main causes of implant failure. The Moss Miami SI System was used in our patient. The screw of this system was locked with an inner nut and an outer coil around the tail of the screw. The locking procedure was completed in 2 stages. The inner nut was first locked and then the outer coil was used to finish the locking procedure. This complex procedure might be prone to manipulation faults.

Plain films after the index surgery showed that the position of the screws was appropriate. The rods were not rotated in the correct orientation. The curve of the rod was directed outward. The angles of the transverse plane of these screws on 1 side were not accordance. A contradictory force on the monoaxial screws of 1 rod is theoretically possible. Abnormal stress on the screws affects the stability of the instrumentation. In our case, the rod gradually migrated into the spinal canal and caused cauda equina syndrome. There was no evidence of metallosis or infectious erosion. Improper manipulation and an imperfect screw design were considered to be the reasons for instrumentation failure. One year after the second operation, the rod had slightly shifted. Ideally, a revision surgery should have been performed when we noticed that the rod had slightly shifted, but the patient declined. This migrated rod finally became a hazard to the spinal cord. Radiological follow-up is important to prevent this complication.

In conclusion, the reasons for implantation failure vary. Infection, metallosis, and fusion failure are common reasons, but improper manipulation is also possible. If implantation failure is suspected, the situation should be addressed or the patient followed up more closely.
